# Current state-of-the-art and potential future therapeutic drugs against COVID-19

**DOI:** 10.3389/fcell.2023.1238027

**Published:** 2023-08-25

**Authors:** Ailong Sha, Yi Liu, Haiyan Hao

**Affiliations:** ^1^ School of Teacher Education, Chongqing Three Gorges University, Chongqing, China; ^2^ School of Biology and Food Engineering, Chongqing Three Gorges University, Chongqing, China; ^3^ School of Environmental and Chemical Engineering, Chongqing, China

**Keywords:** anti-SARS-CoV-2 drug, animal model, screening, viral load, viral titer

## Abstract

The novel coronavirus disease (COVID-19) continues to endanger human health, and its therapeutic drugs are under intensive research and development. Identifying the efficacy and toxicity of drugs in animal models is helpful for further screening of effective medications, which is also a prerequisite for drugs to enter clinical trials. Severe acute respiratory syndrome coronavirus-2 (SARS-CoV-2) invades host cells mainly by the S protein on its surface. After the SARS-CoV-2 RNA genome is injected into the cells, M protein will help assemble and release new viruses. RdRp is crucial for virus replication, assembly, and release of new virus particles. This review analyzes and discusses 26 anti-SARS-CoV-2 drugs based on their mechanism of action, effectiveness and safety in different animal models. We propose five drugs to be the most promising to enter the next stage of clinical trial research, thus providing a reference for future drug development.

## 1 Introduction

The novel coronavirus disease (COVID-19) is an acute respiratory infectious disease caused by severe acute respiratory syndrome coronavirus-2 (SARS-CoV-2), after infection, it will cause serious damage to the body, and there may be a variety of sequelae. At present, it is still in a global pandemic, with outbreaks in many countries and regions and a high incidence trend (Contini et al., 2023; Lippi et al., 2023; Pagani et al., 2023). As of 30 September 2022, the cumulative number of confirmed cases and deaths worldwide has exceeded 600 million and 6 million, respectively (World Health Organization, 2022). So far, the research and development of COVID-19 therapeutic drugs is mainly carried out from three aspects: new use of old drugs, convalescent plasma therapy, and new drug development (Liu et al., 2020; McKee et al., 2020; Hillary and Ceasar, 2023). Its treatment mainly focuses on two main aspects: targeting the coronavirus itself or regulating the human immune system. The coronavirus itself has many targets for drug design, and the immune response increasingly shows its advantages in anti SARS-CoV-2 infection (Rotondo et al., 2022; Polatoğlu et al., 2023). Among the open new treatment methods, many scientific and effective methods have also been found, such as using computer drug design technology or structure detection to conduct macromolecular docking analysis between drugs and SARS-CoV-2 target spots, which can detect whether they have affinity with SARS-CoV-2. In addition, drug similarity characteristics can also be evaluated to determine whether it has the potential to become a standard for drugs. If the standard is established, it can further detect the absorption, distribution, metabolism, excretion, and toxicity of potential candidate drugs, that is, using human or humanized tissue functional proteins as “drug targets”, *in vitro* research techniques combine with computer simulation methods. Studying the interaction between drugs and biophysical, biochemical barrier factors in the body, namely, drug ADMET characteristic detection, which not only determines their safety but also provides reference for pharmacokinetic characteristics (Wang J. et al., 2022; De Arthur et al., 2022; Azzouzi et al., 2023). After completing the above online detection and evaluation, the applications of experimental animals and animal model experiments become extremely important. The research and development of new drugs are closely related to the applications of experimental animals and animal models. The study of drug efficacy and toxicity in experimental animals and animal models is an important guarantee to promote the development of new drugs, which is helpful to explore the bioavailability of drugs or to develop new therapies together with other drugs. So far, many drugs have been evaluated in COVID-19 animal models. Therefore, this review is to screen out the promising COVID-19 drugs from a variety of anti-SARS-CoV-2 drugs that have been tested in animal pharmacology, based on the effectiveness of drug target action, drug safety and the effective use of related traditional Chinese patent medicines and simple preparations, to provide a reference for the researchers who urgently need to carry out clinical trials. Based on this review, over time, it may bring the long-awaited COVID-19 specific therapeutic drugs to the patients with COVID-19.

## 2 The infection mechanisms of SARS-CoV-2, the action mechanisms of anti-SARS-CoV-2 drugs, and pre-clinical animal models

### 2.1 The infection mechanisms of SARS-CoV-2

**FIGURE 1:**
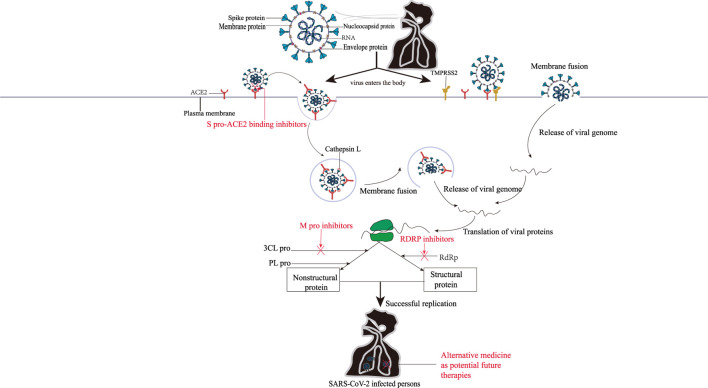
The mechanisms of SARS-CoV-2 entry into cells and the action mechanisms of COVID-19 therapeutic agents. There are two ways for SARS-CoV-2 to enter the cells. Left: SARS-CoV-2 was introduced by Cathepsins L if the TMPRSS2 is insufficient or a SARS-CoV-2–ACE2 complex does not encounter TMPRSS2; Right: TMPRSS2 is present, and the SARS-COV-2-ACE2 complex binds to the cell. Subsequently, the viral genome was then released by membrane fusion and replicated in the cells under the action of 3CL pro, PL pro, RdRp, and other enzymes. The red font represents anti-SARS-CoV-2 drugs that inhibit the corresponding targets.

As shown in Figure 1, the routes of SARS-CoV-2 infecting cells are all related to the spike protein (S pro), which exists on the surface of virus in the form of a trimer, and each S pro includes a head S1 subunit and an S2 subunit. Among them, the head subunit S1 containing a receptor binding domain (RBD), which exists on the surface of SARS-CoV-2, can bind with human receptor—angiotensin -converting enzyme 2 (ACE2) to form the RBD-ACE2 complex. The S2 subunit is close to the viral membrane and is responsible for mediating the fusion between the virus and the host cell membrane. The two heptapeptide repeat domains (HR1, HR2) of the S2 subunit are highly conserved, and they both play a vital role in the formation of the six-helix bundle (6-HB) core structure by virus fusion (Wang Q. et al., 2020; Wang X. et al., 2020). The entry of SARS-CoV-2 into cells usually requires two S pro cleavage events: one at the junction of the S1 and S2 subunits, and the other at the S2’ site—inside the S2 subunits (Jackson et al., 2022). When SARS-CoV-2 invades cells, transmembrane serine proteinase 2 (TMPRSS2) on the surface of target cells can cleave S pro and produce an unlocked fusion-catalysis form on the cell surface, which helps viruses enter host cells quickly (Yadav et al., 2021). If TMPRSS2 is insufficient or fails to meet the SARS-CoV-2-ACE2 complex, the latter will be internalized into the endolysosome through clathrin-mediated endocytosis, and the S2’ cleavage will be performed by Cathepsin L (Karthika et al., 2021). If TMPRSS2 is present, S2 ‘cleavage occurs directly on the cell surface. After that, the fusion peptide (FP) is exposed after the S2’ site is cleaved. The dissociation of S1 and S2 induces the conformational change of S2, which pushes FP forward into the target membrane and initiates the membrane fusion to form the fusion pore. SARS-CoV-2 RNA is released into the cytoplasm of the host cell through the fusion pore for encapsulation and replication, and the infection process is completed (Koch et al., 2021; Takeda, 2022).

After entering the cell, SARS-CoV-2 RNA relies on the host ribosome to produce two multi-proteins (PP1A and PP1ab), which will release 16 non-structural proteins under the action of Papain-like protease (PL Pro) and master protease (3 chymotrypsin like protease, 3CL pro, also known as M pro) (Yan and Wu, 2021). Among them, 11 cleavage sites of the nonstructural protein nsp4-nsp16 can be specifically recognized and cleaved by the nonclassical H41-C145 catalytic dimer between domains I and II of 3CL Pro. The nsp4-nsp16 is not only responsible for genome replication and transcription, but also plays a role in other critical life processes, such as protein translation, cleavage, modification, and nucleic acid synthesis (Ullrich and Nitsche, 2020). The rest of the genome will encode four major structural proteins [S pro, Nucleocapsid (N protein), Membrane protein (M protein), envelope protein (E protein)] and nine accessory proteins (Orf3a, Orf3b, Orf6, Orf7a, Orf7b, Orf8, Orf9b, Orf9c, and Orf10) of SARS-CoV-2 (Shannon et al., 2020; Zhou et al., 2020). These viral proteins are involved in the formation of SARS-CoV-2 virus particles, which allow SARS-CoV-2 to replicate in the body, interact with host proteins to regulate the immune responses, and then infect other cells.

### 2.2 The action mechanisms of anti-SARS-CoV-2 drugs

As shown in Figure 1, the binding of S pro on the surface of SARS-CoV-2 with ACE2 is the first prerequisite for SARS-CoV-2 to enter the host cell. Therefore, blocking the binding of S pro with ACE2 can be the primary means for the research and development of anti-SARS-CoV-2 drugs (Jin et al., 2020). The fusion-mediated proteins TMPRSS2 and Cathepsin L play a key role in the infection process when S pro binds to ACE2. Therefore, the host cell protease inhibitor based on membrane fusion can also prevent the entry of SARS-CoV-2 (Chen et al., 2021). During replication, the SARS-CoV-2 genome is translated into proteins by host cellular mechanisms, including the non-structural proteins M pro, PL pro, helicase and RNA-dependent RNA polymerase (RdRp). Under the action of RdRp, SARS-CoV-2 RNA generates a full-length antisense negative strand template, and then synthesis of subgenomic mRNAs. Translation of subgenomic mRNAs will produce viral structural proteins that are recognized and packaged with positive-strand RNA to form mature viral particles those are released outside the cell to continue infecting other cells. Whereas, because PL pro is highly similar to human proteases, PL pro-targeted inhibitors may generate a non-specific inhibitory phenomenon, therefore, drugs which selectively inhibit 3CL pro activity are more efficacious (Bugatti et al., 2022; Chen et al., 2022). Thus, the key protease in the replication cycle of those viruses is also an attractive target for anti-SARS-CoV-2 drugs. In addition, combining traditional Chinese medicine and conventional therapy in the treatment of COVID-19 is beneficial to increase the cure rate, shorten the course of disease and reduce the mortality rate of patients (Bestion et al., 2022). Herbal medicine and preparations can also play antiviral, anti-inflammatory, and immunomodulatory roles in COVID-19 therapy through multi-component, multi-pathway, and multi-target pathways (Wang and Yang, 2021; Yang et al., 2022), and have gained unique advantages in COVID-19 therapy, which is expected to make more significant progress.

### 2.3 The various pre-clinical animal models for testing therapeutics against SARS-CoV-2

The pre-clinical animal model is conducive to further clarifying the pathogen transmission, pathogenesis, histopathological changes of COVID-19, finding the effective antiviral drug, and accelerating the development of vaccines. Based on this, the COVID-19 animal model came into being. For example, the cynomolgus macaque and ferrets can simulate human mild COVID-19 (Rockx et al., 2020a; Shi et al., 2020); Human ACE2 (hACE2) transgenic mice can simulate human severe, lethal COVID-19 Bao et al. (2020); And Golden Syrian hamsters can be used to study the pathogenesis of moderate-to-severe COVID-19 in humans (Muñoz-Fontela et al., 2020; Bestion et al., 2022; Qiu et al., 2022). Summarize the animal models currently used in COVID-19, which can be mainly divided into non-human primates, rodents and other mammals, with different limitations and advantages, which are listed in Table 1.

**TABLE 1:** Comparison of the advantages and disadvantages of COVID-19 animal models before clinical trials.

Types			Advantages	Disadvantages
Non-human primate	Rhesus monkey Deng et al. (2020), Lu et al. (2020), Yu et al. (2020)		Being closely related to humans and susceptible to infection with viruses	Expensive price, difficult operation, and limited research has been conducted with this model
	Cynomolgus monkey Rockx et al. (2020b); Lu et al. (2020)		Being closely related to humans	The price is expensive, there are significant individual differences in animals, and the number of animals is small
	African green monkey Woolsey et al. (2021)		The virus replication level is high, and respiratory symptoms are severe, which can develop into a severe patient	Expensive price, difficult operation, and limited research quantity
	Ordinary velvet monkey Lu et al. (2020)		It is closely related to humans, and its population is large, making it easy to operate	The infection rate of COVID-19 is relatively low compared with other animal models
Rodents	Wild rodents	Adenovirus mediated hACE2 wild-type mice Zost et al. (2020), Zhang et al. (2021)	Humanized ACE2 receptor with good fidelity	High modeling costs, and fewer cases of severe illness or death in research subjects
		Syrian hamster Warner et al. (2017); Chan et al. (2020)	Low research cost, and possible occurrence of some clinical symptoms	Lack of research subjects for severe illness or death
		Mouse infection model based on mouse adapted virus strain and recombinant virus strain Wang et al. (2020c), Dinnon et al. (2020), Gu et al. (2020)	It can simulate some clinical pathological changes, and has good repeatability	Mutant or recombinant strains may cause immune escape
	Genetically modified rodents	K18-hACE2 mice Oladunni et al. (2020), Zheng et al. (2021)	Has susceptibility characteristics, and can exhibit some clinical manifestations and pathological changes	The symptoms of fatal encephalitis differ greatly from those of human infections
		HFH4-hACE2 mice Jiang et al. (2020)		
		CAG-hACE2 mice Tseng et al. (2007), Yoshikawa et al. (2009)	Pulmonary viruses can replicate efficiently, and exhibit pathological changes during the acute phase of SARS-CoV-2 infection	The mortality rate is relatively high
		MACE2-hACE2 mice Bao et al. (2020); Sun et al. (2020b)	Can simulate partial clinical manifestations and pathological changes in lung tissue	Individuals may have significant differences in hACE2 expression
Other mammals	Ferret Kim et al. (2020); Park et al. (2020)		Susceptible to viruses	No significant virus replication in the lungs, lacking clinical symptoms
	Chinese tree shrew Xu et al. (2020)		Small size, low breeding cost, and short breeding cycle	Low sensitivity to infection
	Cats, dogs Shi et al. (2020), Halfmann et al. (2020), Sit et al. (2020)		Large body size, and susceptibility to infection	Difficult to operate, not a conventional mode animal
	Mink Shuai et al. (2020)		Easily infected with viruses, the infection mechanism is very similar to that of humans	High case fatality rate

## 3 Anti-SARS-CoV-2 drugs

**FIGURE 2:**
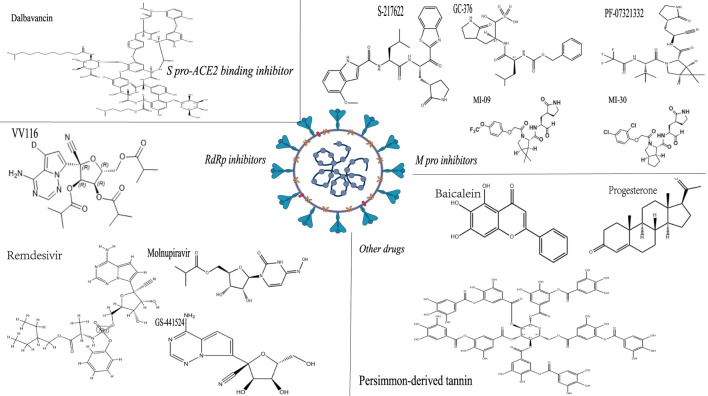
The chemical structure of some anti-SARS-CoV-2 drugs. Note: The structure shown in the figure is the corresponding medicinal chemistry structure, the upper left corner is the drug name, and the italics are the drug classification.

The efficacy, toxicity and chemical structure of different anti-SARS-CoV-2 drugs in animal models are shown in Table 2; Figure 2.

**TABLE 2:** The efficacy and toxicity of anti-SARS-CoV-2 drugs in animal models.

Drug	Inhibition rate of viral loads	Inhibition rate of virus titers	IC50	EC50	Mode of administration	Toxicological experiments	SARS-CoV-2 variants	References
S pro-ACE2 binding inhibitor								
Dalbavancin	41.3%	−	−	12 nM	i.p	−	−	Wang et al. (2021a)
	50%	33.3%			i.v			
REGN10987/REGN10933	99%	−	−	−	i.v	−	D614	Baum et al. (2020), Uraki et al. (2022)
	−	28.4%	−	−	i.p		BA.1	
COV2-2196/COV2-2130	−	28.3%	−	−	i.p	−	D614G	Meng et al. (2022)
S309	−	14.5%	−	−			BA.1.1	
C135-LS/C144-LS	90%	−	3 ng/mL	−	i.v	−	−	Schäfer et al. (2021), Van Rompay et al. (2021)
LY-CoV-016	−	−	0.6–3.2 μg/mL	−	i.v	+	−	Dougan et al. (2021), Wang et al. (2022b)
P5-22	−	50%	0.0072 ug/mL	−	i.p	−	BetaCoV	Zou et al. (2022)
P14-44			0.7 ug/mL	−				
F61/H121	70%	−	−	−	Intranasal administration	−	B.1.1.529	Lu et al. (2022)
S-20-1	30%	−	8.14 μM	−	Intranasal administration	+	P.1(γ)	Xue et al. (2022)
EKL1C	−	93%	0.003 μmol/L	−	Intranasal administration	−	B.1.351, B.1.1.7	Xia et al. (2019)
M pro inhibitor								
GC-376	75.5%	−	−	0.5–3.4 μM	i.p	+	−	(Joaquín Cáceres et al., 2021; Fu et al., 2020; Ma et al., 2020; Sacco et al., 2020)
PF-07321332	77%	78%	−	−	i.g	−	B.1.351、B.1.617.2、B.617.2.332	Owen et al. (2021), Abdelnabi et al. (2022)
ALG-097111	55%	71%	7 nM	200 nM	i.g	−	−	Vandyck et al. (2021)
MI-09	40%	−	−	0.86 μM	i.g	+	−	Qiao et al. (2021)
	70%				i.p			
MI-30	56%	−	−	0.54 μM	i.p			
S-217622	−	70%	0.013 μM	0.29 μM	i.g	−		(Uraki et al., 2022; Unoh et al., 2022)
RdRp inhibitor								
Remdesivir	67%	57%	0.77 uM	−	i.v	−	−	Williamson et al. (2020)
Molnupiravir	50%	99%	3.3 μM	−	i.g	−	B.1.529.1	Abdelnabi et al. (2021), Rosenke et al. (2021), Rosenke et al. (2022), Takashita et al. (2022)
GS-441524	−	50%	0.7 μM	−	i.p	+	−	Li et al. (2022)
VV116	46%	50%	−	0.35 μM	i.g	+	−	Xie et al. (2021)

Note: Inhibition rate of viral loads (%) = (viral loads in the animal control group infected solely with SARS-CoV-2, viral loads in the treatment group of the COVID-19, animal model after treatment with therapeutic drugs)/viral loads in the animal control group infected solely with SARS-CoV-2 × 100%; Inhibition rate of virus titers (%) = (virus titers in the animal control group infected solely with SARS-CoV-2, virus titers in the treatment group of the COVID-19, animal model after treatment with therapeutic drugs)/virus titers in the animal control group infected solely with SARS-CoV-2 × 100%; intraperitoneal injection, i.p.; intravenous injection, i.v., gastric administration, i.g.; in the toxicology experiment column, “+” represents that the toxicology experiment has been conducted, “−” represents that the toxicology experiment has not been conducted; In the SARS-CoV-2, variants column, “−” represents that the variant information is not mentioned.

### 3.1 S pro-ACE2 binding inhibitor

#### 3.1.1 Dalbavancin

As an essential receptor of coronavirus infection, ACE2 has also been found to bind to S pro with high affinity in SARS-CoV-2, which is considered as a therapeutic target for COVID-19 patients (Sharma et al., 2022). Some old drugs can bind to SARS-CoV-2, and studies have found that as an approved lipid peptide antibiotic, dalbavancin can inhibit the binding of ACE2 to SARS-CoV-2 S pro, thereby exerting anti SARS-CoV-2 activity. The *in vitro* experiments have shown that the EC50 of dalbavancin was 12 nM, which could obviously inhibit the combination of ACE2 and SARS CoV-2 S pro. In addition, 400 μM dalbavancin also inhibited the activity of Cathepsin L by 40%, and the EC50 was greater than that of dalbavancin in inhibiting the binding of ACE2 and SARS-CoV-2 S pro. *In vivo*, the K18-hACE2 mouse model and the rhesus monkey model can be used to evaluate the anti-SARS-CoV-2 effect of dalbavancin. The COVID-19 models of K18-hACE2 mice were intraperitoneally injected with 130 mg/kg of dalbavancin to observe the therapeutic effect, and the viral loads in the lung tissue of the mice were reduced by 29.4% and 41.3% on day 1 and day 3, respectively. The Rhesus monkey model of COVID-19 was intravenously injected with the same dose of dalbavancin, and the viral loads and viral titers in the lung tissue were significantly lower (decreased by about 50% and 33.3% respectively) than those in the control group on the 7th day (Wang G. et al., 2021). The results indicated that dalbavancin could significantly inhibit SARS-CoV-2 infection in both mouse and rhesus monkey COVID-19 models. However, due to the differences of the experimental animal models, it is not yet possible to prove the optimal therapeutic effect by this experiment, and the therapeutic effect of different drug administration modes in the same animal model can be evaluated subsequently. In addition, dalbevancin toxicology experiments were conducted on rats and dogs, and it was found that single dose toxicity showed clinical adverse reactions (including decreased activity, slow breathing, hair erection, and seizures) after administration, which subsequently weakened over time (Committee for Medicinal Products for Human Use, 2014). In repeated dose studies, it can induce systemic toxicity, with the main target organs being the kidneys, liver, and blood system, and the distribution of toxicity varies among different tissues. However, its toxic exposure is significantly lower than human expectations and is not critical for the available clinical safety data. Therefore, the clinical dosage is 1,000 mg intravenously on the first day and 500 mg intravenously 1 week later, indicating overall good safety characteristics (Committee for Medicinal Products for Human Use, 2014; Dmitri, 2014).

#### 3.1.2 REGN10987/REGN10933

REGN-COV2, namely, REGN10987/REGN10933, a combination of the monoclonal antibodies imdevimab and casirivimab, which has been proved to significantly reduce the risk of hospitalization or death in high-risk groups of COVID-19, and reduce the viral load (RECOVERY Collaborative Group et al., 2021; Weinreich et al., 2021). REGN-COV2 can target non-overlapping epitopes on SARS-CoV-2 S pro and bind to SARS-CoV-2 S pro specifically. Studies have evaluated the anti-SARS-CoV-2 activity of the REGN-COV2 antibody combination in rhesus monkeys and golden hamsters models. For example, the viral loads almost disappeared in the rhesus animal model administered with REGN-COV2 intravenously at a dose of 50 mg/kg before infected by SARS-CoV-2, and the ones were also significantly inhibited in nasopharyngeal and oral swabs with the increasing concentration of SARS-CoV-2. The inhibitory effect was REGN-COV2-dose-dependent. The viruses were rapidly removed in the nasopharynx and oral cavity of the animals treated with 25 mg/kg or 150 mg/kg of REGN-COV2 after infected by SARS-CoV-2, the viral loads in the oral swabs decreased more significantly, and the efficacy at different doses was the same. The golden hamster can be used as an infected animal model to simulate the severe type of patients with COVID-19. After treatment with different concentrations of REGN-COV2 (50, 5, and 0.5 mg/kg) for 7 days, the weight of golden hamsters in different treatment groups reduced in a dose-dependent manner, and both gRNA and SgRNA in lung tissue reduced by more than 99% (Baum et al., 2020). In an other study, the anti-SARS-CoV-2 effect of this antibody combination was also evaluated, which was administered intraperitoneally to Syrian hamsters, and the virus titers in the turbinate and lung tissues was found to decrease by 30.6% and 28.4%, respectively (Uraki et al., 2022). The above results indicated that the anti-SARS-CoV-2 activity of REGN-COV2 has been confirmed by a variety of animal models, and the therapeutic and preventive effects of this antibody combination on upper respiratory tract infection in animals were better. In addition, toxicological experiments have shown good safety. Phase II clinical trials have shown that REGN-COV2 can be administered at low doses and subcutaneous injections, and there were no adverse safety signals or dose-related safety findings (Committee for Medicinal Products for Human Use, 2021; Portal-Celhay et al., 2022).

#### 3.1.3 COV2-2196/COV2-2130, S309

According to the World Health Organization (WHO), from August 26 to 26 September 2022, the Omicron variant still accounted for 99.9% of the reported SARS-CoV-2 gene sequence worldwide (World Health Organization, 2022). It is divided into six different subpopulations, among which BA.1 has been found to be less sensitive to some monoclonal antibodies by a variety of *in vitro* experiments. Uraki et al. (2022) evaluated the therapeutic effects of the combination of COV2-2196/COV2-2130, S309, and other monoclonal antibodies approved by the US Food and Drug Administration (FDA) on the BA. 1 variants. The test drugs were administered intraperitoneally to Syrian hamsters 1 day after infection with SARS-CoV-2, and their nasal turbinates and lung samples were collected for virus titration 4 days later. In the D614G variants, the viral titers in hamster turbinates after treatment with COV2-2196/COV2-2130 and S309 decreased by 45.1% and 3.6%, respectively, and those in lung tissues by 28.3% and 14.5%, respectively. In the BA. 1 variants, the viral titers in hamster turbinates after treatment with COV2-2196/COV2-2130 and S309 decreased by 9.2% and 10.4%, respectively, and in lung tissues by 41.2% and 12.3%, respectively. Further evaluation of COV2-2196/COV2-2130 single antibody and combination used in the BA1.1(NC929) sub-variant showed that they both had no inhibitory effect on the BA1.1. Analysis of the causes, the D614G variant and the BA.1 variant had the same replicative efficiencies in human nasal epithelial cultures, but the viral titers of Omicron variant was lower than the Delta variant in lower respiratory organs and lung tissue cells (Meng et al., 2022). The reason why COV2-2196/COV2-2130 has no inhibitory effect on the BA.1 subvariant NC929 needs further study, maybe the response to other SARS-CoV-2 variants can be determined. And caution should be exercised in using this antibody alone or in combination in infected patients.

#### 3.1.4 C135-LS/C144-LS

Both C135-LS and C144-LS monoclonal antibodies have been shown to have anti-SARS-CoV-2 activity with IC50 of 3 ng/mL *in vitro*. The test drug was administered by intravenous infusion (40 mg/kg, 12 mg/kg) in the rhesus monkey COVID-19 model. The inhibition of SARS-CoV-2 was observed in all dose groups on the first day, the RNA copy numbers of the nasal swabs of the treatment groups decreased the most on the fifth day, and the inhibition rate of SARS-CoV-2 increased with the increase of drug concentration, while the drug efficacy decreased with the increase of the days of infection. The oropharynx swab showed the most significant decrease in RNA copy numbers on the 3rd day, and then maintained its persistent inhibition, the inhibition rate of viral RNA copy numbers in lung tissue was more than 90%, and increased with the increase of drug concentration. In addition, (Schäfer et al., 2021) also tested C135-LS and C144-LS in mouse and hamster models, and found that administration of drugs at the early stage of infection would be more helpful to reduce lung viral load. That is, the combined antibody of C135-LS/C144-LS can effectively inhibit SARS-CoV-2 replication in the pre-infection stage and prevent disease progression (Van Rompay et al., 2021).

#### 3.1.5 LY-CoV-016

LY-CoV-016, a recombinant human monoclonal neutralizing antibody, also known as Etesevimab, can effectively block all five RBDs binding to ACE2 receptor, its IC50 value is 0.6–3.2 μg/mL. LY-CoV-016 was intravenously injected into hACE2 transgenic mice, followed by 5 × 10^5^ TCID50 SARS-CoV-2 was administered intranasally, and the mice were injected again 1 day later. It was found that the viral RNA in the lungs of the mouse was significantly lower than that of the control group. Four of the five mice in the intravenous injection group could not detect the viral RNA 2 days after SARS-CoV-2 infection (Wang F. et al., 2022). In the phase III clinical trial, LY-CoV-016 could reduce the hospitalization rate and mortality of COVID-19 and accelerate the decline rate of SARS-CoV-2 viral load. For adverse reactions, the main manifestations were nausea, rash, dizziness, diarrhea, and hypertension, but varied from person to person (Dougan et al., 2021).

#### 3.1.6 IBI314

Zou et al. (2022) constructed a novel antibody hybrid therapy consisting of p5-22 antibody and p14-44 antibody in a 1:1 ratio and named it IBI314. It can bind to two different RBD, block the interaction between RBD and ACE2 receptor, and show the super-potent neutralization to SARS-CoV-2. The IC50 values of P5-22 and P14-44 were 0.0072 and 0.7 ug/mL *in vitro*, respectively. It showed that P5-22 had more potent antiviral activity than LY-CoV-016. SARS-CoV-2 was induced in Ad5-hACE2-transduced mice by intraperitoneal injection of IBI314 at doses of 2, 10 or 50 mg/kg 1 day after infection. On the third day, the virus titers in the lung tissue of animals in each dose group decreased by 50%, and the pathological changes in the lung tissue were alleviated, which indicated that IBI314 had an noticeable antiviral effect.

#### 3.1.7 F61/H121

Lu et al. (2022) found that two neutralizing antibodies, F61 and H121, showed extensive neutralizing activity against wild and variant SARS-COV-2 strains (including Beta, Delta, and Omicron strains) by intranasal administration. Among them, the F61 can block the binding of viruses to ACE2 by recognizing a linear epitope in the ACE2-RBD binding domain, while the H121 can bind to the ACE2 conformational epitope located on the conserved side of RBD (Barnes et al., 2020). And the combination of F61 and H121(1:1) showed synergistic neutralization due to the binding of different epitopes. The IC50 value of F61/H121 combination to Omicron mutant was 0.13 μg/mL *in vitro* (Qu et al., 2021). *In vivo* studies showed that the F61/H121 combination exhibited significant prophylactic protection against the lethal challenge of Delta and Omicron variants in the COVID-19 model of K18-HACE2 mice by intranasal administration at a dose of 20 mg/kg, and the viral loads in the lung tissue of mice inoculated with the low and high virus inoculation groups decreased by more than 70% and 40%, respectively. In addition, a low dose of F61 or a combination of F61 and H121 could also reduce the viral loads in the lung and brain tissue of animals. These results indicated that both antibodies showed apparent anti-SARS-CoV-2 effects.

#### 3.1.8 S-20-1

The S pro on the surface of SARS-CoV-2 is the major antigen, and it is the critical protein that SARS-CoV-2 binds to the host receptor, mediates the fusion of SARS-CoV-2 with the host membrane, and enters the host body. Therefore, the fusion mechanism of SARS-COV-2 is an essential target for the development of fusion inhibitors (Yuan et al., 2020). The RBD in the S1 subunit and HR1 domain in the S2 subunit of SARS-CoV-2 S pro are the targets of neutralizing antibody (mAb) and pan-coronavirus (CoV) fusion inhibitory peptides, respectively. However, neither mAbs nor peptides can be administered orally. Xue et al. (2022) developed a novel long-acting pan-coronavirus fusion inhibitor, S-20-1, which based on cyclic γ-A-peptide, and could target two sites of SARS-CoV-2 S pro. S-20-1 can effectively inhibit many SARS-CoV-2 variants, such as B. 1.617.2 and B. 1.1.529. The IC50 and CC50 values were respectively 8.14 and 692.7 μM *in vitro* cell test, and showed low cytotoxicity. S-20-1 showed extensive inhibitory activity against a variety of variants such as B.1.1.7, B.1.351, B.1.617.2, and other variants in different cell lines. The anti-SARS-CoV-2 effect was detected in the COVID-19 model of hACE2 transgenic mice by intranasal administration of 60 mg/kg, and it was found that the viral loads in the brain and lung tissues of mice in the treatment group decreased by about 20% and 30%, respectively. At the same time, the mouse model of intranasal administration for 12 days, showed no significant changes in the body weight of the mice and no inflammatory reaction in tissues. This indicates that this inhibitor is safer, and S-20-1 exhibits better oral bioavailability compared to other inhibitory peptides, with a longer half-life. Therefore, it is expected to become a new oral drug against COVID-19 in the future.

#### 3.1.9 EKL1C

In addition to S-20-1, (Xia et al., 2019) designed the HR2 sequence in the S protein of HCoV-OC43 and developed the first universal CoV entry inhibitor EK1 peptide for SARS-CoV-2 infection and S protein mediated membrane fusion. And Zhou et al. (2022) explored that de-pegylated lipopeptide EKL1C showed highly effective inhibitory activity on SARS-CoV-2 S pro mediated membrane fusion, by coupling cholesterol molecules with EK1 peptide for SARS-CoV-2 infection and S pro mediated membrane fusion. In cell assays *in vitro*, EK1C4 prevented SARS-CoV-2 replication in a dose-dependent manner, and the IC50 was 0.003 μmol/L, CC50 values were 10, 13.81 and 8.49 μM in Huh-7, Caco-2 and 293T/ACE2 cells, respectively, and SI value was between 222 and 345. Human ACE2 protein expression (hACE2-TG) mice were given EKL1C intranasally at the dose of 1.5 mg/kg before and after SARS-CoV-2 infection *in vivo*. After 4 days, the viral titers in the lung tissue of the treatment group decreased by more than 93%, and the decrease in the prevention group was even higher. These results indicate that EKL1C can effectively inhibit SARS-CoV-2 infection *in vitro* and *in vivo*. In addition, EKL1C is more resistant to proteolytic enzymes and trypsin than EK1C4.

### 3.2 M pro inhibitors

#### 3.2.1 GC-376

GC-376 has been confirmed to have anti-SARS-CoV-2 activity *in vitro* (Fu et al., 2020; Ma et al., 2020; Vuong et al., 2020). Joaquín Cáceres et al. (2021) evaluated the toxicity and anti-SARS-CoV-2 activity of GC-376 in K18 hACE2 mice. Mice infected with low and high doses of the SARS-CoV-2 virus were treated with an intraperitoneal injection of 20 mg/kg GC-376. There was no significant weight loss and 100% survival rate after 7 days of administration, which served as the evidence that GC-376 had no acute toxicity. After 5 days of treatment, the viral RNA loads in the nasal concha, lung and brain of the mice in the high-dose exposure group decreased by 58.8%, 8.3%, and 72%, respectively, while the ones in the low-dose exposure group decreased by 6.7%, 43.8%, and 75.5%, respectively. These results suggested that GC-376 had a significant therapeutic effect on the mice with different degrees of infection, but the degree of virus inhibition in different tissues was not consistent after treatment. The viral RNA loads in the turbinate did not decreased with the decrease of infection degree, which might be related to the difference in the inhibitory effect of GC-376 on SARS-CoV-2 from different tissue site. Further experimental evaluation is still needed. The lung associated index is one of the hallmarks of assessing the severity of COVID-19, and the viral loads in the brain can cause numerous neurological symptoms and even threaten life (Ellul et al., 2020; Helms et al., 2020). Therefore, reducing the viral loads in the lung and brain is essential for the treatment of COVID-19. In addition, the EC50 values of GC-376 have been reported as 0.5-3.4 µM *in vitro* (Fu et al., 2020; Ma et al., 2020; Sacco et al., 2020), but further investigation on the optimal *in vivo* anti-SARS-CoV-2 concentration of GC-376 is needed. Although this experiment preliminarily concluded that GC-376 had no acute toxicity through the indexes of body weight change and survival rate of the mouse model, but the specific toxicity indicators such as LD50, 95% confidence limit, and safe range *in vivo* needed to be further improved.

#### 3.2.2 PF-07321332

PF-07321332, which is a chemically modified derivative of PF-00835231, also known as nirmatrelvir, can target the main protease of SARS-CoV-2, and inhibit SARS-CoV-2 *in vitro* effectively. In the SARS-CoV-2 MA10 mouse model, the anti-SARS CoV-2 activity of PF-07321332 *in vivo* was evaluated by gavage at doses of 300 or 1,000 mg/kg. On the 4th day, the virus titers in the lung tissues of mice in the two dose groups decreased by 26.5% and 38.8%, respectively (Owen et al., 2021). Abdelnabi et al. (2022) further evaluated the anti-SARS-CoV-2 effect of PF-07321332 on different variants of SARS-CoV-2 in the Syrian hamsters. Syrian hamsters infected with the SARS-COV-2 β variant were treated by gavage (125 or 250 mg/kg twice daily). On the fourth day, the viral RNA copy numbers in hamster lung tissues of the two-dose groups decreased by 15.4%, and 77%, respectively, and decreased by 18.2% and 78% in the viral lung titers, respectively. In conclusion, although the higher dose of PF-07321332 was used in the mouse infection model, the inhibitory effect of PF-07321332 was weaker than that of PF-07321332 in the Syrian hamster model. Therefore, more attention should be paid to its potential scope of action in preclinical evaluation, and further toxicological studies should be conducted to assess its toxicity. In addition, Paxlovid, a compound antiviral drug synthesized by Ritonavir and PF-07321332, has been found to be effective against COVID-19 and has entered phase II/III clinical studies. Paxlovid has been fully approved for use by the UK (Medicines and Healthcare products Regulatory Agency, 2021), EU (European Medicines Ag ency, 2023), Canada (Health Canada, 2022), and the US (FDA News Release, 2023), but its safety is not yet very clear, and after occasional treatment, the virus rebounds and symptom recurs (Akinosoglou et al., 2022).

#### 3.2.3 ALG-097111

Vandyck et al. (2021) evaluated the anti-SARS-CoV-2 effects of ALG-097111 *in vitro* and in the Syrian hamsters. It was found that the EC50, IC50, and SI values of ALG-097111 were 200 nM, 7 nM, and 500, respectively, and the activity of Cathepsin L was inhibited by 50% at 10 μM. After intragastric administration of 200 mg/kg ALG-097111, the RNA copy numbers and virus titers in the lung tissues of hamsters were decreased by 55% and 71%, respectively. However, the bioavailability of ALG-097111 by gavage was low, and its toxicity was not tested.

#### 3.2.4 MI-09, MI-30

By comparing with the existing protease inhibitors, (Qiao et al., 2021) designed two inhibitors, MI-09 and MI-30, which can effectively inhibit the major protease of SARS-CoV-2. No apparent toxicity was observed *in vitro* (CC50 > 500 μM), and the EC50 values of MI-09 and Mi-30 were 0.86 and 0.54 μM, respectively. *In vivo* animal model, no acute toxicity was determined by intravenous injection (40 mg/kg) of MI-09 or MI-30 without rat death, and no significant toxicity was respectively observed for 7 days in the animals after intravenous injection of 6, 18 mg/kg, or intraperitoneal injection of 100, 200 mg/kg. The drug was given by gavage or intraperitoneal injection (50 mg/kg) in the hACE2 transgenic mouse model, and the viral loads in lung tissues were measured on the first day. It was found that the viral loads in the lung tissues of the MI-09 group in the low virus inoculation model mice (the amount of virus vaccination was 2 × 10^6^ TCID50) were decreased by 40% and 65% by gavage and intraperitoneal injection, respectively. After intraperitoneal injection of MI-30 (50 mg/kg), the viral loads in the lung tissues of the model mice were reduced by 54%. The inoculation concentration of SARS-CoV-2 was increased to 5 × 10^6^ TCID50 to simulate moderate SARS-COV-2 infection, and the viral loads in the lungs of the animals were decreased by 5%, 10%, and 20%, respectively, on the first day after MI-09 administration, MI-09 intraperitoneal injection or MI-30 intraperitoneal injection. The viral loads were decreased by 40%, 70%, and 56%, respectively, on the third day, no viral loads were detected in both groups on the fifth day. By comprehensive comparison, MI-09 and MI-30 administered by intraperitoneal injection were more effective than by gavage, and they can significantly inhibit the replication of SARS-CoV-2 under moderate virus infection, and improve the lung injury caused by SARS-CoV-2.

#### 3.2.5 S-217622

S-217622 is an inhibitor of SARS CoV-2 M pro protease, of which the IC50, EC50 values *in vitro* were 0.013, and 0.29 μM, respectively. The anti-SARS-CoV-2 effect of S-217622 was evaluated by gavage in mice, and it was found that the viral titers in mice were reduced by 70% after S-217622 treatment, and the effect was dose-dependent (Unoh et al., 2022). In addition, in the Syrian hamsters, the viral titers of SARS-CoV-2 in the turbinates were decreased by 18.3 and 9.9 times on the 2nd and 4th day, respectively, while S-217622 did not significantly inhibit the virus titers in the animal’ turbinates on the 7th and 14th day after administration. It is speculated that S-217622 may have the best therapeutic effect in the early stage of SARS-CoV-2 infection (Uraki et al., 2022).

### 3.3 RdRp inhibitors

#### 3.3.1 Remdesivir

Several treatments have been approved by the FDA for patients infected with COVID-19, Remdesivir is one of them. Remdesivir has been shown to effectively inhibit SARS-CoV-2 *in vitro*, its IC50 value is 0.77 uM, so it has attracted much attention. Williamson et al. (Williamson et al., 2020) tested the anti-SARS-CoV-2 efficacy of Remdesivir in the rhesus monkey model at a daily intravenous dose of 5 mg/kg. On the 7th day, it was found that the viral load in the lung tissue of the animal in the Remdesivir group was significantly lower than that in the control group, with the inhibition rate about 67%, and the viral titer in bronchoalveolar lavage (BAL) was significantly reduced. Twelve hours after the first Remdesivir administration, the viral titer in the BAL of the animals in the treatment group was about 100 times lower, and the inhibition rate could be roughly calculated as 57%. There was little detectable infectious virus in BAL, and in addition, animals treated with Remdesivir had milder lung lesions. In conclusion, the effect of Remdesivir was the best in the early stage, so it should be considered as early as possible in clinical treatment.

#### 3.3.2 Molnupiravir

MK-4482, an oral antiviral nucleoside analogue, has been licensed for use in high-risk adult patients for anti-SARS-CoV-2 emergency treatment (Jayk Bernal et al., 2022a; FDA, 2022), but has not been used as a routine therapeutic agent. Its anti-SARS-CoV-2 activity was evaluated *in vitro* with an IC50 of 3.3 μM (Takashita et al., 2022). Molnupiravir at a concentration of 250 mg/kg was administered by gavage 12 h and 2 h before infection, and 12 h after infection in the *Mesocricetus auratus* model. It was found that the copy numbers of SARS-CoV-2 RNA in the lung tissues of the hamsters model were doubled after treatment, indicating that the infection of SARS-CoV-2 in the lung tissue could be reduced by 50%, and the numbers of virus titers were reduced by 50%, suggesting that MK-4482 has a strong inhibitory effect on SARS-COV-2 replication in animals (Rosenke et al., 2021).

Molnupiravir also showed potent antiviral activity against the emerging SARS-CoV-2 variant. Abdelnabi et al. (2021) administered Molnupiravir at a concentration of 200 mg/kg by gavage to a hamster model, and found that the RNA copy numbers in the lung tissues of hamsters infected with SARS-CoV-2 B.1-G, B.1.1.7, and B.1.351 decreased by 20%, 25%, and 35%, respectively, and the viral titers decreased by 42%, 45%, and 60%, respectively. These results suggested that Molnupiravir did have a significant inhibitory effect on the emerging SARS-CoV-2 variant. Another study analyzed the inhibitory effects of different variants of the SARS-CoV-2 by Syrian hamster administration of Molnupiravir at a dose of 250 mg/kg by gavage, and the results showed that on the fourth day, Molnupiravir inhibited the viral RNA loads in the oral swabs of the hamsters model by 12.5%, 6.7%, and 17%, respectively, the viral loads inhibition rates in the lung tissues of the hamsters model were 9%, 14.4%, and 100%, respectively, the virus titers in lung tissues were decreased by 48.8%, 47.4% and 100%, respectively (Rosenke et al., 2022). It was shown that MK-4482 had an inhibitory effect on different variants of SARS-CoV-2, with the most potent inhibition on the latest variant, Omicron, and is most likely effective on inhibiting the subsequent emergence of new variants of SARS-CoV-2. In addition, Molnupiravir was found to be safe and well tolerated through Phase I adaptability test, and had a strong protective effect on people who have not been vaccinated with COVID-19 vaccine (Khoo et al., 2021; Fischer et al., 2022; Khoo et al., 2023; Jayk Bernal et al., 2022b).

#### 3.3.3 GS-441524/VV116

Remdesivir can prevent serious adverse reactions caused by respiratory failure in patients infected with COVID-19. It has good efficacy in both early and severe patients, which has entered clinical trials and is the only COVID-19 treatment drug approved by the US Food and Drug Administration (Beigel et al., 2020; Gottlieb et al., 2022). However, the efficacy of Remdesivir may be affected by genetic factors, resulting in differences in the efficacy of clinical trials (Wang LY. et al., 2020). Both GS-441524 and VV116, as derivatives of remdesivir, have obvious anti SARS CoV-2 effects. Li et al. (2022) found that the Remdesivir metabolite GS-441524 had a strong anti-SARS-CoV-2 effect, with an IC50 value of 0.7 μM in VeroE6 cells, which was stronger than that of Remdesivir (IC50 of 1.35 μM), however, the anti-SARS-CoV-2 effect of Remdesivir was more substantial in Calu cells, that is, the relative potency of GS-441524 and Remdesivir was related to cell type. The AAV-hACE2 mice were injected intraperitoneally with 25 mg/kg GS-441524 1 day before infection and lasted for 8 days, the virus titers in the lung tissues of the mice were decreased by 50% after administration on the second day. The results suggested that GS-441524 had less cytotoxicity and toxicity *in vivo* than Remdesivir, and could effectively inhibit SARS-CoV-2 infection, which was expected to become a promising drug for the treatment of COVID-19.

VV116, also a derivative of Remdesivir, has a pronounced anti-SARS-CoV-2 effect. *In vitro* cell experiment, the EC50, CC50, and SI values were 0.35, 280.19 μM, and 800, respectively. VV116 was administered intragastrically at doses of 25, 50, or 100 mg/kg in the hACE2-transduced mice model, respectively. On the second day, the levels of SARS-CoV-2 RNA and virus titer in the lung tissue of each dose group of mice were detected, and decreased by more than 25% and 40%, respectively. On the fifth day, they decreased by 46% and more than 50%, respectively, the area and extent of inflammatory lesions in lung tissues were also significantly improved. The IC50 of VV116 was 0.67 μM (the one of Remdesivir was 1.35 μM), the maximum tolerated single dose was 2 g/kg and 1 g/kg in rats and beagle dogs, respectively. And no adverse reactions were observed in the 14-day repeated dosing study at doses of 200 mg/kg and 30 mg/kg, respectively, oral bioavailabilities were 80% and 90%, respectively, and the metabolites were distributed in the preferred target (included the intestine, lungs, kidneys, liver, heart, and brain) of SARS-CoV-2 *in vivo* (Xie et al., 2021). In addition, VV116 has a shorter sustained clinical recovery time and higher safety compared to nirmatrelvir-ritonavir (Cao et al., 2023). In conclusion, VV116 has a strong anti-SARS-CoV-2 effect and high safety, which may be a new therapeutic drug for COVID-19. GS-441524 and VV116 are both derivatives of Remdesivir, and have strong anti SARS-CoV-2 effect, or can be used together to achieve better curative effect.

### 3.4 Alternative medicine as potential future therapies

Alternative medicine as potential future therapies is shown in Table 3.

**TABLE 3:** Alternative medicine as potential future therapies.

Drug	Inhibition rate of viral loads	Inhibition rate of virus titers	IC50	EC50	Mode of administration	Toxicological experiments	SARS-CoV-2 variants	References
Lianhuaqingke	9%	−	684.2 ug/mL	−	i.g	+	−	Wang et al. (2021b)
Baicalein	96.8%	−	−	1.69 μM	i.g	+	−	Song et al. (2021)
Persimmon derived tannin	25%	23%	−	−	i.g	+	−	Furukawa et al. (2021)
Human placenta hydrolysate	−	30%	−	−	i.v	−	−	Kim et al. (2021)
Progesterone	13%	−	−	−	i.p	+	−	Yuan et al. (2022)
ALD-R491	−	−	64.9 nM	−	i.g	−	−	Li et al. (2021)

Note: intraperitoneal injection, i.p.; intravenous injection, i.v.; gastric administration, i.g.; in the toxicology experiment column, “+” represents that the toxicology experiment has been conducted, “−” represents that the toxicology experiment has not been conducted; In the SARS-CoV-2, variants column, “−” represents that the variant information is not mentioned.

#### 3.4.1 Lianhuaqingke

The IC50 and TC50 (median toxic concentration) values of Lianhuaqingke were 684.2 and 972.2 ug/mL, respectively, and the SI value was greater than 1. In the COVID-19 model of hAEC2 transgenic mouse, the viral load in the lung tissue was reduced by 9% after 5 days of treatment with a dose of 14.67 g/kg of Lianhuaqingke solution by gavage (Wang M. et al., 2021). The Lianhuaqingke mainly exerted its anti-SARS-CoV-2 effect by inhibiting virus replication and regulating immune response. Although its inhibitory effect was not strong, its safety was relatively high, or it could be used as an anti-SARS-CoV-2 drug by combining with other medicines to enhance its antiviral effect.

#### 3.4.2 Baicalein

Baicalein in *Scutellaria baicalensis* Georgi can inhibit the injury of VeroE6 cells induced by SARS-CoV-2 in a dose-dependent manner. Baicalein can directly protect the cells from the injury induced by SARS-COV-2 when the concentration of baicalein was 0.1–0.3 μM, and the EC50, CC50 values of baicalein were 1.69 μM, 0.2 mM, respectively, the SI value was more significant than 19. When baicalein was administered intragastrically to the hACE2 mice infected with SARS-COV-2, the viral loads in the lungs of the mice were reduced by 96.8% and 90.9% on the third and fifth day, respectively, these results suggested that baicalein could significantly inhibit the replication of SARS-CoV-2 in lung tissue. Further toxicity tests showed that a single dose of baicalein did not show apparent signs of hepatotoxicity and nephrotoxicity, and there was no noticeable accumulation of baicalein in multiple doses. It is suggested that both single and multiple doses of baicalein were safe and well tolerated, and Baicalein mainly played its role by inhibiting virus replication and regulating immunity, so baicalein was a potential drug for the treatment of COVID-19 (Song et al., 2021). In addition, baicalin is the main phase II metabolite of baicalein, and its crystal form β absolute bioavailability is higher than its crystal form α, more promising for treatment (Song et al., 2021).

#### 3.4.3 Persimmon-derived tannin

It has been shown that persimmon-derived tannin has an anti-virus effect on many kinds of viruses. Furukawa et al. (2021) also found that it had strong anti-SARS-CoV-2 activity, and was administered by gavage in the COVID-19 model of Syrian Hamster, it was found that the virus titers and viral loads in the lung tissues of hamsters decreased by 23% and 25% on the third day, respectively. And by cohabiting hamsters treated with persimmon-derived tannins with infected Syrina hamsters, it was found that the former could effectively prevent infection, which indicated that persimmon-derived tannin had a strong anti-SARS-CoV-2 effect, and the effect of that pretreated with persimmon-derived tannin was stronger. In addition, persimmon derived tannins can not only inhibit the SARS-CoV-2 titer and inflammatory response in the lungs to improve the pathogenesis of COVID-19, but also induce SARS-CoV-2 aggregation, which may be an important link in its antiviral mechanism. However, this experiment has not shown that the specific components of persimmon-derived tannins has anti-SARS-CoV-2 activity, therefore, the detailed mechanism that persimmon-derived tannins aggregated SARS-CoV-2 needs to be revealed. Persimmon-derived tannins are mainly polyphenolic compounds polymerized from catechins, including epicatechin (EC), epicatechin gallate, epigallocatechin, and epigallocatechin-3-gallate (EGCG). Jang et al. (2020) found that EGCG, an active component also presented in green tea, had prominent anti-SARS-CoV-2 activity *in vitro*, therefore, specific persimmon-derived tannin monomers can be studied separately. In addition, the toxicity test of persimmon-derived tannin was not carried out in this experiment, and it needed to be further supplemented to verify its safety.

#### 3.4.4 Human placenta hydrolysate

Kim et al. (2021) found that the human placenta hydrolysate (hph) had significant anti-SARS-CoV-2 activity with low cytotoxicity, and the CC50 > 100%, EC50 > 19.42%, SI>5.1% for hph. Ferrets were injected intravenously with hph (4 mL/animal) after being infected with SARS-CoV-2, it was found that the viral titers in the nasal cavity, lung, and concha of ferrets were decreased by about 50%, 30%, and 40%, respectively, and the level of type I IFN in ferrets was significantly increased after treatment with hph, that was, the viral loads of SARS-CoV-2 were decreased. The results suggested that hph had a significant anti-SARS-CoV-2 effect. However, the optimal antiviral concentration and mechanism of action are not yet clear, and further research is needed.

#### 3.4.5 Progesterone

It has been shown that the severity of COVID-19 in the female is lower than that in the male (Clark et al., 2020). Yuan et al. (2022) found that progesterone can improve the severity of COVID-19 in the Syrian hamster model, after Syrian hamsters were infected with SARS-CoV-2, they were intraperitoneally injected with progesterone at a dose of 1, 3, or 5 mg/kg. On the 7th day, the weight losses of all hamsters were decreased, and the viral RNA copy numbers in lung tissue were decreased by 13%, with the increase of progesterone dosage. The main action mechanism of progesterone was its ability to inhibit the excessive release of pro-inflammatory cytokines and SARS-CoV-2 replication. Therefore, progesterone may become a potential therapeutic drug for COVID-19 in the future.

#### 3.4.6 ALD-R491

It has been found that extracellular vimentin is a kind of attachment factor that promotes SARS-CoV-2 to enter human cells. As a structural protein, vimentin is widely expressed in interstitial-derived cells, and is a potential new anti-SARS-CoV-2 target with possible use in blocking SARS-CoV-2 infection (Amraei et al., 2022). Li et al. (2021) found that ALD-R491 could affect general cellular processes and specific cellular functions (such as reducing endocytosis, endoplasmic transport, and extracellular release; increasing the microbicidal ability of macrophages; enhancing the activity of regulatory T cells) related to SARS-COV-2 infection by altering the physical properties of vimentin filaments. Thus, COVID-19 can be treated by host-directed antiviral and anti-inflammatory actions. The EC50 and CC50 values of ALD-R491 were 0.036, and 10 μM, respectively. In the virus infection experiment, the IC50 was 64.9 nM, and SI was 154 when the multiplicity of infection was 50. ALD-R491 was administered intragastrically to SARS-CoV-2-infected rats. After 6 h, the concentration of ALD-R491 in lung tissue was maintained at about 1 μM, while its effective concentration *in vitro* was 0.1 μM, the results suggested that intragastric administration was effective *in vivo*. The anti-SARS-CoV-2 effect of ALD-R491 was evaluated by the SARS-CoV-2 mouse-adapted (MA) model in the form of intragastric administration at doses of 3, 10, or 30 mg/kg, respectively, and prophylactic administration was found to be ineffective. While the medium and high doses of ALD-R491 showed noticeable therapeutic effects under the condition of therapeutic administration, the virus loads and virus titers in the lung tissues of the animals were decreased, but the specific effective dose range was not clear and needed to be further evaluated in a variety of model animals to determine the effective dose range.

## 4 Discussion

Due to the instability of SARS-CoV-2 RNA, it is still in continuous evolution. Considering the action mechanisms of anti-SARS-CoV-2 drugs from the aspects of cell infection, entry and replication are beneficial to the development of therapeutic drugs for COVID-19. In this paper, based on the mechanisms of action and targets of different anti-SARS-CoV-2 drugs, considering the efficacy and safety of various medications, it is believed that among the S pro ACE2 binding inhibitors, C135-LS/C144-LS and EKL1C show the most substantial inhibition effect on COVID-19 compared with REGN10987/REGN10933, which have entered the clinical trial. EKL1C shows more vital anti-SARS-CoV-2 activity comparing with the minimum inhibition concentration *in vitro*. EKL1C is a lipopeptide-based pan-coronavirus fusion inhibitor, which has significantly stronger anti-protease hydrolysis and thermal stability, and is very effective against SARS-CoV-2 Omicron variant infection. However, the pharmacological experiments of this drug are insufficient at present, extensive animal models are still needed for pharmacological experiments. Among M pro inhibitors, compared with PF-07321332 and S-217622 in phase II/III clinical trials, the EC50 values of GC-376 and MI-09 are higher, which means their safety is higher, but short of efficacy. Alternatively, they can be combined with other drugs to combat SARS-CoV-2 infection. Among RdRp inhibitors, the efficacy of Remdesivir derivative VV116 is not necessarily the best compared with the drugs entering the clinical trial (such as Galidesivir, Favipiravir, and Molnupiravir), but its IC50 is the smallest, for example, its IC50 is only 1/5 that of Molnupiravir. The results showed that the drug concentration needed to achieve 50% inhibition of the pathogen was lower, and the oral bioavailability was higher, which can be further supplemented for concentration research. For other drugs, baicalein has the strongest inhibitory effect on SARS-CoV-2, and its EC50 is higher than that of S-217622 in clinical trials, that is, it is safer, and it is expected to enter clinical research after improving its toxicological experiments.

Regarding the five drugs discussed above, GC-376 acting on M pro of SARS-CoV-2 can inhibit virus replication and infection of new cells, and since there is no M pro in the human body, it is less likely that drugs targeting M pro will cause side effects. EKL1C, a lipopeptide-based pan-coronavirus fusion inhibitor, has more extensive antiviral activity than the monoclonal antibody currently in development, targeting more conserved HR1 or HR2 domains, and can be transported and stored at room temperature. Therefore, these two drugs are the most promising therapeutic agents and can be evaluated in clinical trials.

In summary, screening prospective drugs can be considered from the following aspects: firstly, the effectiveness of target effects; The second is the safety testing of drugs, which considers safety hazards based on their effectiveness; Thirdly, the effective use of traditional Chinese patent medicines and simple preparations. In addition, when conducting pharmacological experiments before clinical trials, due to the diversity of SARS-CoV-2 variants that are prevalent internationally, it is necessary to fully consider the effective inhibition degree of the drug on different variants, and to use multiple animal models, especially those with very similar infection mechanisms and symptoms to humans. In terms of drug action gradient, it is necessary to combine the pharmacokinetics of the drug with the characteristics of animal models to contrast to human specificity.
